# Gender Differences and Role of Pregnancy in the History of Post-Surgical Women Affected by Tetralogy of Fallot

**DOI:** 10.1371/journal.pone.0049729

**Published:** 2012-12-11

**Authors:** Luciano Daliento, Lucia Dal Bianco, Francesco Bagato, Eleonora Secco, Berardo Sarubbi, Elisa Mazzotti, Barbara Bauce, Giulio Rizzoli

**Affiliations:** 1 Cardiology Unit, Department of Cardiac, Thoracic and Vascular Surgery, University of Padua Medical School, Padua, Italy; 2 Cardiology Unit, University of Naples Medical School, Naples, Italy; 3 Adult Cardiac Surgery Unit, Department of Cardiac, Thoracic and Vascular Surgery, University of Padua Medical School, Padua, Italy; Sapienza University of Rome, Italy

## Abstract

**Background:**

The aim of this study was to describe gender differences in patients operated on for TOF and to define the impact of pregnancy in late post-surgical follow-up in women.

**Methods:**

In this research, we studied 145 patients after correction of TOF: 66 male, 79 women, 41 of which reported history of 68 pregnancies, means age 37±10 years, age at operation 7±8 years, mean duration of post-surgical follow-up 30±7 years. Selected variables were compared according to sex and according to history of pregnancy with statistical tests.

**Results:**

Men had more severe hemodynamic impairment and a higher number of cardiac reoperations than females. 41% of patients had at least one complication during pregnancy; there were 16 (67%) abortions and 39 (74%) Caesarian delivers; the recurrence of congenital heart defect was 10%. After pregnancy, there was a shift from first to second functional class: unique pregnancy determined no differences in term of morpho-functional ventricular features compared to nulliparous, but they complained fatigue and palpitation and echocardiographyc dysfunction. Left ventricular dysfunction and QRS duration at ECG were independent predictors of ventricular arrhythmias in all patients.

**Conclusions:**

There were no gender-specific differences in patients operated on for TOF using ventriculotomy. Pregnancy is an event in these patients at risk for the newborn, in terms of miscarriage, prematurity, and recurrence of birth defects, and for the mother in terms of ventricular dysfunction and electrical instability. At least a single pregnancy does not appear to significantly modify the natural history of post-surgical patients operated on for TOF.

## Background

Successful surgery of TOF, dating back to 50 years ago, has produced the largest group of survivors among the GUCH (Grow-up Congenital Heart) population, affected from complex congenital heart diseases [1], [2].

Many studies have observed that ventricular dysfunction and arrhythmias are the determinants of late postoperative morbidity and mortality [3], [4], [5].

There are no significant gender differences in the incidence of TOF [6] and there is large number of female patients who have reached the reproductive age. However the role of gender differences [7] and of one or several pregnancies on patients' clinical history, quality of life, morbidity and survival are poorly known.

We questioned if our pregnant TOF operated patients developed in their medical history factors conditioning their morbidity more frequently and/or prematurely compared with nulliparous women.

The purpose of this study was to collect clinical and instrumental data of a group of women operated on for correction of TOF, who had one or more pregnancies and compare their data with those of nulliparous women and with male group.

The three groups were operated concomitantly with similar techniques (right ventriculotomy), operative age and duration of post-surgical follow-up and our aim was to determine whether there are gender differences in clinical development of this post-surgical population and whether pregnancy can change it.

## Materials and Methods

### TOF population in post-surgical follow-up

185 patients (87 men and 98 women) operated on for correction of tetralogy of Fallot between 1960–2010 had a post-surgical follow-up of 5502 patients-years (mean 30±8 years), during whose 13 patients (7% mortality) died (7 men and 6 women) with a mean age of 46.02±8.63 years (M: 45.80±7.26 years; W: 46.24±9.81 years, p = 0,732), after a post-surgical follow-up of 29.30±9.52 years (M: 30.85±6.14 years; W: 27.75±11.77 years, p = 0,548).

Heart failure (7 patients (53.85%), 4 men and 3 women) and sudden death (4 patients (30.77%), 1 man and 3 women) were the main causes of death. One patient died because of pulmonary embolism, one patient in a car accident. One male patient was transplanted at the age of 45 years.

Only 145 survived patients (66 men and 79 women) were enrolled in this study because they are routinely visited at our outpatient clinic and were controlled during the last calendar year (Table 1).

**Table 1:** Patients enrolled.

	ALL	MEN	WOMEN	NO PREGNANT	PREGNANT	P
**Patients**	145	66	79	38	41	
	(100%)	(45.52%)	(54.48%)			p = NS
			(100%)*			p* = NS
				(48.10%)*	(51.90%)*	
**Mean Age (yrs)**	37.72±10.40	37.32±10.27	38.07±10.49	35.85±10.54	40.16±10.00	
						p = NS
						p* = NS

(*)  =  Referred only to women population (N = 79).

Table 2 summarizes data regarding patients that underwent direct surgical correction or after palliation (Blalock-Hanlon anastomosis).

**Table 2:** Surgical data.

	ALL	MEN	WOMEN	NO PREGNANT	PREGNANT	P
**Palliation**	57	27	30	13	17	
	(100%)	(47.37%)	(52.63%)			p = NS
			(100%)*			p* = NS
				(43.33%)*	(56.67%)*	
**Mean Age at palliation**	2.11±3.11	2.41±3.97	1.83±2.02	1.16±1.84	2.34±2.01	
**(yrs)**						p = NS
						p* = NS
**Mean Age at total correction**	7.61±8.25	7.51±7.70	7.70±8.67	5.68±6.03	9.56±10.20	
**(yrs)**						p = NS
						p* = NS
**Mean duration post surgical follow-up**	30.11±7.66	29.81±7.14	30.35±8.06	30.17±7.094	30.60±8.17	
**(yrs)**						p = NS
						p* = NS

(*)  =  Referred only to women population (N = 30).

The primary objective of clinical and instrumental follow-up was to collect for each patient demographic data, functional capacity, the clinical and morpho-functional status of the cardiac chambers with arrhythmic risk stratification. Other concomitant data were: educational level, employment status, marital status and practice of physical activity or sports; presence of symptoms (dyspnea at rest, fatigue, palpitations, fainting and syncope), functional class (NYHA and Ability Index); drugs, presence of cardiac device (pacemaker/ICD), comorbidities with particular attention to anxious-depressive syndromes, cardiac reoperations and extra-cardiac surgery.

Female patients were given a questionnaire with reference to obstetric history. We investigated about date and age of each pregnancy/abortion/interruptions/birth, about complications during pregnancy (heart failure, arrhythmias, worsening of clinical status, use of medication), NYHA class and Ability Index pre-and post-pregnancy were also collected as well as the type of delivery (EuToch/dystocia, futures/pre-term), the number of children and the possible recurrence of birth defects or congenital heart disease.

For arrhythmic risk stratification, patients were classified at low risk if 24 hours ECG Holter monitoring documented simple ventricular arrhythmias (Lown class not more than 4) and at high risk if episodes of sustained and/or non-sustained ventricular tachycardia were recorded (severe arrhythmias). Patients who presented atrial fibrillation have also been reported.

The following electrocardiographic parameters were considered: rhythm, QRS complex duration [ms], presence of right bundle branch block, mean duration of QT interval [ms] and corrected QT interval [ms], the mean QT interval dispersion [ms].

According to recommendations of American and European Societies of Ecocardiography [8] the following parameters were calculated: end-diastolic volume (EDV) and ejection fraction of the left and right ventricle (LVEF–RVEF). Semilunar and A-V valves status, incompetence, residual obstruction or intra-cardiac shunts were evaluated by the CW-Doppler and PW-Doppler.

### Ethical aspects

The protocol of this study (regarding the clinical follow-up of pregnancies women) was revisited and approved by Ethical Committee of our hospital (Comitato Etico per la Pratica Clinica, Azienda Ospedaliera di Padova). Informed consent from all particiants had not been obtained because the study was retrospective and didn't involve the patients themselves. The study was a statistical analysis about clinical information. The study is completely anonymous and the ethics committee approved the consent procedure.

### Statistical analysis

It was performed by means of STATA (Stata Corp. 2009. *Stata Statistical Software: Release 11*. College Station, TX: StataCorp LP) [9].

Data were reported in tables comparing males vs. females and pregnant vs. nulliparous females.

Categorical and class variables were summarized as number of patients and frequency percentages and compared by means of two-way Fisher exact test (categorical variables) or Chi-square (class variables).

Continuous variables were summarized by the number of patients, mean and standard deviation and compared with the Student t test.

Coded variables were similarly summarized and compared with the Kruskal-Wallis non parametric test.

Repetitive events (pregnancy, reoperations) were analyzed segmenting the longitudinal history into multiple epochs, each ending at the time of recurrence of the morbid event and their probability was reported with the Nelson Aalen cumulative failure curve and compared with the log rank test.

A multivariate logistic analysis of the echocardiographic predictors of severe arrhythmia including gender and pregnancy status was also performed.

## Results

### Obstetric history

**Figure 1:**
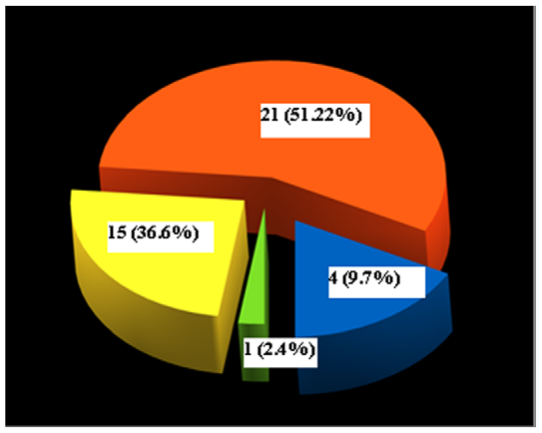
Demographic representation of women population based on pregnancies history.

79 patients were female, 38 women (48.10%) were nulliparous, 41 (51.90%) reported a history of at least one pregnancy, for a total of 68 pregnancies. 21 patients (51.22%) reported one pregnancy, 15 patients (36.59%) had two pregnancies, 4 patients (9.76%) three pregnancies. One patient (2.44%) had 5 pregnancies (Fig. 1).

**Figure 2:**
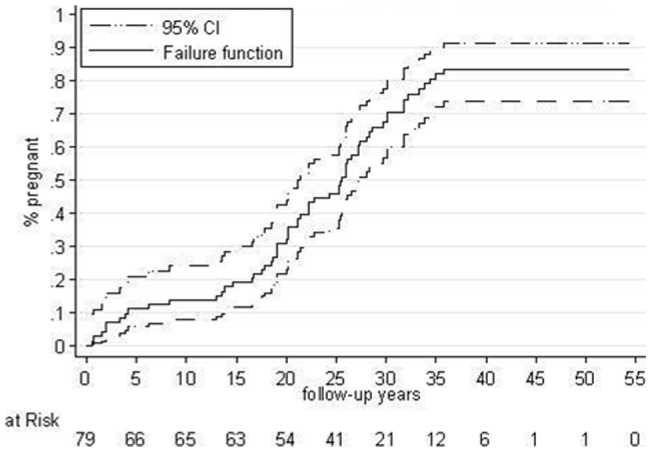
Post-operative pregnancy failure function.

The mean age of patients at first pregnancy was 26.58±4.37 years, at the second was 29.53±4.37 years, at the third was 33.82±6.17 years (Fig. 2).

34 of 41 patients (82.93%) completed the pregnancy without complications: 23 (56.10%) had a single birth, 10 (24.39%) two births and one patient (2.44%) three births, for a total of 46 pregnancies at term without complications (68%).

The remaining 22 pregnancies (32%) were complicated: 2 (3%) pregnancies were characterized by bleeding and placental disruption; 4 (6%) deliveries were preterm (described as <37 gestational week); 16 (23%) pregnancies failed (5 abortions, 10 miscarriages, 1 fetus born died).

**Figure 3:**
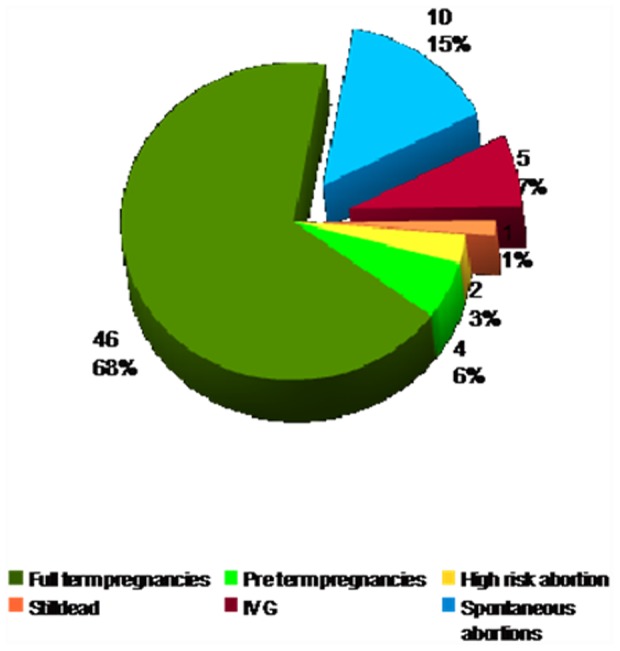
Global analysis of 68 pregnancies.

Pregnancy terminations (miscarriage and abortion) were present in 12 women (15.19%): eight of them (10.13%) had one pregnancy termination, four of them (5.06%) had two events, for a total of 16 events. The age of patients at first pregnancy termination was a mean of 28.88±4.69 years, the mean age at second event was 29.80±7.24 (Fig. 3).

Among pregnancy terminations (16 events in total), 10 of them (62,5%) were miscarriages, occurred in 8 patients (10,13%): 7 patients (18,86%) had one event, one patient (1,27%) had 3 events.

14 childbirths (26.42%) were natural, 39 by Caesarean section (73.58%). The recurrence of congenital heart diseases was 10.35% (3/53: 2 were inter-ventricular septal defects, one was uni-ventricular heart).

### Gender and equality differences

#### Demographic and status parameters

There have been no statistically significant differences between men and women regarding age, education in various degrees (about 30% have a high school diploma, 15% attended University, reaching a bachelor's degree in 10% of cases), civil status and practice of physical activity or sports.

Women are employed especially in part-time jobs (15% vs. 3%, p = 0.006); men in full-time jobs (34% vs. 22%, p = 0.01). There have been no differences between patients with a history of pregnancy and nulliparous women regarding age, educational level, employment status, practice of physical activities/recreational sports.

There was a prevalence of pregnancy among married woman (67% vs. 33%, p = 0.02).

#### Clinical parameters

Women complained more frequently fatigue (45% vs. 28%, p = 0.05) and dyspnoea during physical exercise (60% vs. 36%, p = 0.038); however, more females than men belong to functional class Ability Index I (p = 0.08). There were no significant differences for the NYHA and Ability Index classes. Asthenia (p = 0.003) and palpitations (p = 0.02) were more frequently reported by patients with a history of pregnancy. Cardio-active drugs were used by 37% of patients, without gender differences.

Table 3 shows data about the presence of heart devices, as PM and/or ICD. There are no statistically significant differences among the groups; however males underwent ICD implant at younger age than women (p = 0.0001).

**Table 3:** PM and ICD.

	ALL	MEN	WOMEN	NO PREGNANT	PREGNANT	P
**PM (II)**	3	1	2	2	0	
	(2.07%)	(0.69%)	(1.38%)			p = NS
			(2.53%)*			p* = NS
				(2.53%)*	(0.00%)*	
**ICD (II)**	8	5	3	2	1	
	(5.52%)	(3.45%)	(2.07%)			
			(3.80%)*			p = NS
						p* = NS
				(2.53%)*	(1.28%)*	
**Age of ICD implantation**	33.71±11.65	28.43±10.92	44.28±1.11	44.28±1.11	–	P<0.0001 p* = –

(*)  =  Referred only to women population (N = 79).

The women had more comorbidities than men (Table 4).

**Table 4:** Comorbidity.

	ALL	MEN	WOMEN	NO PREGNANT	PREGNANT	P
**Other**	50	19	31	16	15	
**patology (II)**	(34.48%)	(13.10%)	(21.38%)			p = 0.0001
			(39.24%)*			p* = NS
				(20.25%)*	(18.99%)*	
**Anxious-**	10	2	8	3	5	
**depressive**	(6.90%)	(1.38%)	(5.52%)			p = 0.03
**Sindrome**			(10.12%)*			p* = NS
**(II)**				(3.80%)*	(6.32%)*	

(*)  =  Referred only to women population (N = 79).

No statistically significant differences were reported regarding pharmacological treatment, presence of comorbidity or device between women with a history of pregnancy and nulliparous women.

**Figure 4:**
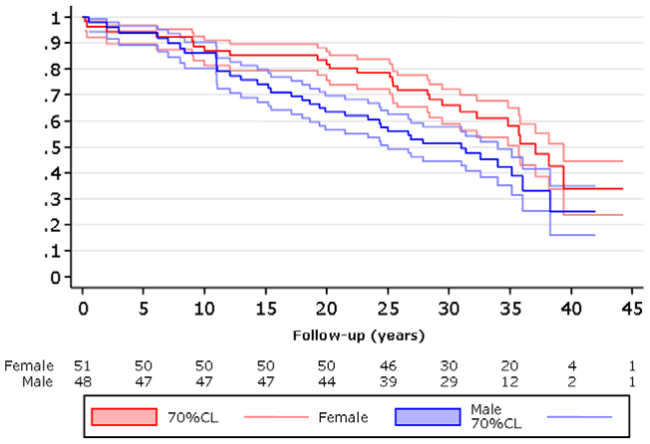
Reoperation-free survival according to sex.

Cardiac reoperations were more frequent (26 vs 16, p = 0,04) and earlier (Fig. 4) in men than in women.

On the contrary extra-cardiac surgery (appendectomy, cholecystectomy, gynecological surgery, trauma) was more frequent in women compared with men (23% vs. 14%, p = 0.08).

### Non invasive diagnostic exams

#### ECG at the last follow-up

There was a significant gender difference regarding the QRS duration, greater in men than in women (163±27 ms vs. 149±26 ms, p = 0.017). There have been no statistically significant gender differences for morphology of QRS complex including right bundle branch block (complete or incomplete), mean duration of QTc (482±42 vs 457±32, p = 0.09) and mean duration of QT dispersion (60±20 vs 51±12 ms, p = ns). There have been no significant differences for any ECG parameter between patients with a history of pregnancy and nulliparous (Table 5).

**Table 5:** ECG analysis at the last follow-up.

	ALL	MEN	WOMEN	NO PREGNANT	PREGNANT	P
**ECG (N)**	78	38	40	18	22	
	(100%)	(48,72%)	(51,28%)			p = NS
			(100%)*	(45,00%)*	(55,00%)*	p* = NS
**Mean duration**	156,75±27,41	163,47±27,27	149,13±25,52	150,08±24,23	148,41±26,44	
**QRS (ms)**	(N = 64)	(N = 34)	(N = 30)	(N = 13)	(N = 17)	p = 0,017
						p* = NS
**RBBi**	4	2	2	1	1	
	(5,33%)	(2,13%)	(2,13%)			
	(N = 75)	(N = 38)	(5,41%)	(2,70%)	(2,70%)	p = NS
			(N = 37)	(N = 16)	(N = 21)	p* = NS
**RBB**	68	34	34	15	19	
	(90,67%)	(36,17%)	(36,17%)			
	(N = 75)	(N = 38)	(91,89%)	(40,54%)	(51,35%)	p = NS
			(N = 37)	(N = 16)	(N = 21)	p* = NS
**Mean duration**	439,53±49	446,76±56,85	431,53±36,91	435,78±47,56	427,70±22,85	
**QT (ms)**	(N = 40)	(N = 21)	(N = 19)	(N = 9)	(N = 10)	p = NS
						p* = NS
**Mean duration**	469,92±38,32	482,31±42,05	457,54±29,37	453,17±31,92	461,29±26,42	
**QTc (ms)**	(N = 26)	(N = 13)	(N = 13)	(N = 6)	(N = 7)	p = 0,09
						p* = NS
**Mean duration**	56,21±16,92	60,29±19,74	51,88±11,84	51,25±13,64	52,50±9,68	
**QTd (ms)**	(N = 33)	(N = 17)	(N = 16)	(N = 8)	(N = 8)	p = NS
						p* = NS

(*)  =  Referred only to women population (N = 79).

#### Echocardiographic data

The quantity echocardiographic data of left and right ventricle are summarized in Tables 6 and 7 respectively.

**Table 6:** Left ventricular size and function (LV EDV  =  Left Ventricular End-Diastolic Volume. LV EF  =  Left Ventricular Ejection Fraction.

	ALL	MEN	WOMEN	NO PREGNANT	PREGNANT	P
**LV EDV**						p = 0.0001
(ml/m2)	59,01±16.28	65.55±19.11	53.42±10.57	52.51±9.51	54.25±11.39	p* = NS
	117	43	74	37	37	
Normal EDV	(80.69%)	(29.66%)	(51.03%)			p = 0.0001
(≤75 ml/m2)			(93.67%)*	(46.84%)*	(46.84%)*	p* = NS
Moderately enlarged LV	15	14	1	0	1	
(87–96 ml/m2)	(10.34%)	(9.65%)	(0.69%)			p = 0.0001
			(1.27%)*	(0.0%)*	(1.27%)*	p* = NS
**LV EF (%)**	58.45±5.67	56.97±6	59.69±5.06	60.5±4.89	58.93±5.09	p = 0.003
						p* = NS
Normal EF	127	54	73	36	37	
(≥55%)	(87.58%)	(37.24%)	(50.34%)			p = 0.07
			(92.41%)*	(45.57%)*	(46.84%)	p* = NS
Moderately reduced EF	5	4	1	0	1	
(44–30%)	(3.45%)	(2.76%)	(0.69%)			p = NS
			(1.27%)*	(0.0%)*	(1.27%)*	p* = NS

(*)  =  Referred only to women population (N = 79).

**Table 7:** Right ventricular size and function. RV EDA  =  Right Ventricular End-Diastolic Area. RV EFA  =  Right Ventricular Ejection Fraction Area.

	ALL	MEN	WOMEN	NO PREGNANT	PREGNANT	P
**RV EDA**	35.96±9.91	39.62±10.06	32.30±8.27	30.87±7.15	33.67±9	p = 0.0002
(cm2)						p* = NS
Normal EDA	10	0	10	7	3	
(≤28 cm^2^)	(6.90%)	(0.00%)	(6.90%)			p = 0.0001
			(12.66%)*	(8.86%)*	(3.80%)*	p* = NS
Moderately enlarged RV	29	11	18	7	11	
(33–37 cm^2^)	(20.00%)	(7.59%)	(12.41%)			p = NS
			(122.78%)*	(8.86%)*	(13.92%)*	p* = NS
Severely enlarged RV	35	24	11	6	5	
(≥38 cm^2^)	(66.20%)	(16.55%)	(7.59%)			p = 0.01
			(13.92%)*	(7.59%)*	(6.33%)*	p* = NS
						
**RV EFA (%)**	34.54±7.63	33.89±7.22	35.09±7.91	36.66±8.38	33.63±7.15	p = NS
						p* = 0.08
Normal EFA	96	45	51	27	24	
(≥32%)	(66.20%)	(31.03%)	(35.17%)			p = NS
			(64.56%)*	(34.18%)*	(30.38%)	p* = NS
Moderately reduced EFA	9	6	3	1	2	
(18–24%)	(6.21%)	(4.14%)	(2.07%)			p = NS
			(3.80%)*	(1.27%)*	(2.53%)*	p* = NS
Severely reduced EFA	2	1	1	0	1	p = NS
(≤17%)	(1.38%)	(0.69%)	(0.69%)			p* = NS
			(1.27%)*	(0.0%)*	(1.27%)*	

(*)  =  referred only to women population (N = 79).

The aortic valve was continent more frequently in women (24.4% vs. 19.5); 3 males underwent to aortic valve replacement with prosthesis. Women with a history of pregnancy had a more severe degree of tricuspid regurgitation (p = 0.007). Regarding other Doppler parameters, we considered trans pulmonary gradient (28±16 mmHg in men, 24±13 mmHg in women, 23±14 mmHg in nulliparous, 24±12 mmHg in multiparous) and residual shunt (6.2% in both sexes); there was no significant sex difference and within pregnant or nulliparus woman.

### Severe arrhythmias

39 of 145 patients (26.9%) had a history of severe ventricular arrhythmias, 20 men (13.79%) and 13 pregnant women (8,97%) and 6 nulliparous (4.14%). 15 (10.34%) patients suffered from atrial fibrillation.

Severely arrhythmic patients had a significantly larger number of re-hospitalization (p = 0.001, OR 3.183), need of ICD implantation (p = 0,005) and therapy (p = 0,002, OR = 3,128; b-blockers: p = 0,001, OR = 4,200; ACEi: p = 0,002, OR = 4,492; ASA: p = 0,022, OR = 6,606), increased number of palliative interventions (p = 0.061) and reoperations (p = 0.006), especially for pulmonary (p = 0,035) or aortic valve replacement (p = 0,020).

**Figure 5:**
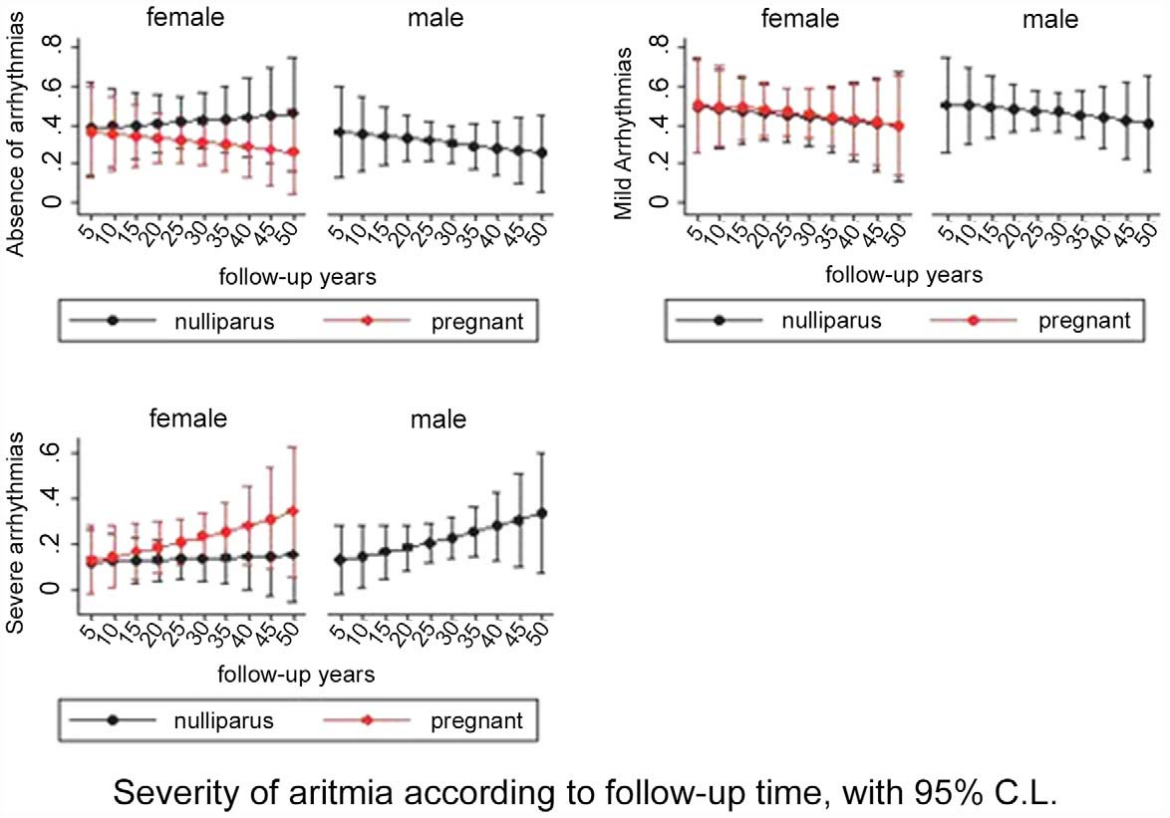
Time related arrhythmia development according to sex of the patient and pregnancy.

These patients in comparison with patients without severe arrhythmias had a longer duration of QRS complex (p = 0.004, OR 1.029) and at Holter electrocardiogram showed a larger numbers of ventricular extrasystoles (p = 0.006, OR 11.818). Echocardiography showed larger end-diastolic volume of left ventricle (p = 0.007, OR 1.029) an lower ejection fraction of right ventricle (p = 0.004, OR 0.916) with a major number of severe ventricular dysfunction, (p = 0.008, OR 2.322). The incidence of severe arrhythmias increased with follow-up time In pregnant women at a higher pace as in males and nulliparous women although the differences were no significant (Fig. 5).

**Figure 6:**
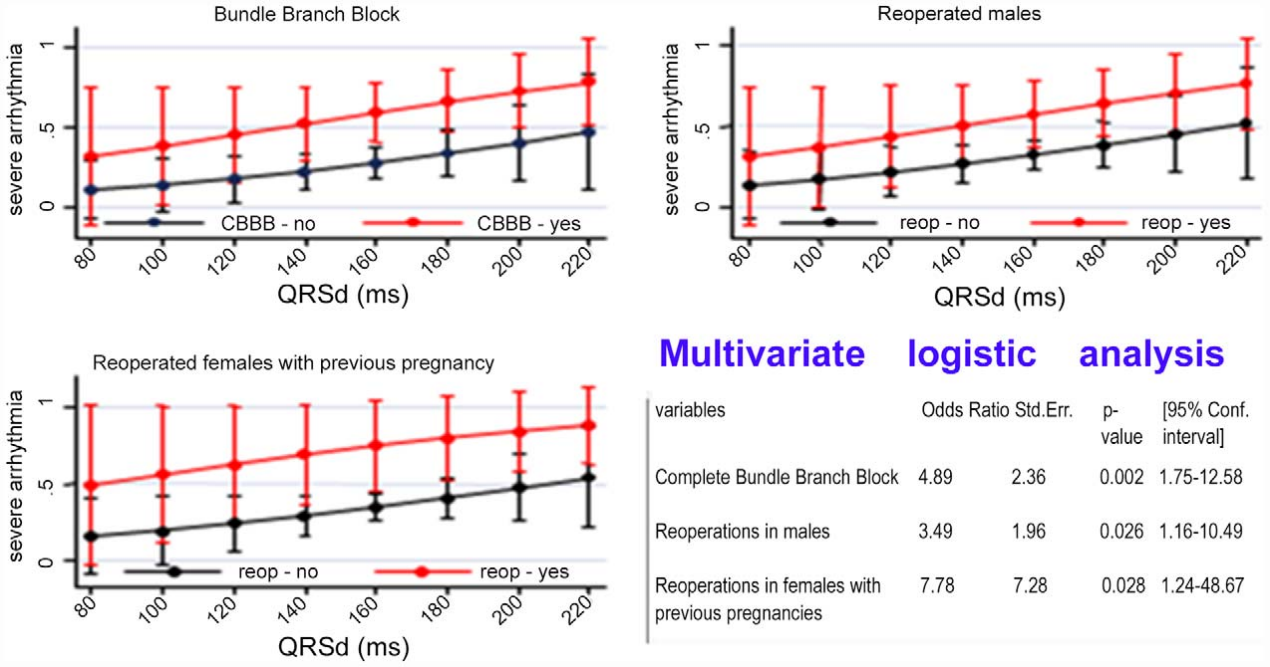
Probability of severe arrhythmia according to results of multivariate analysis.

The probability of severe arrhythmias increased from 21% to 52% in presence of complete RBBB, from 23% to 46% in males who had cardiac reoperations and from 24% to 64% in women with history of pregnancy who had cardiac reoperations. The predictive margins of severe arrhythmias in sex males and females, were showed in Fig 6.

### Pre-post pregnancy modifications

If we consider the successful pregnancies, 83% of patients, had NYHA functional class I at last follow-up pre-pregnancy, 17% had NYHA class II, no patient was in NYHA class III or IV, with a mean of 1.17±0.38. At first follow-up after-delivery, 62% of women remained in class I, 34% in class II and 4% in class III. with a mean of 1.42±0.56. This change was statistically significant (p = 0.0002). The same behavior was seen for Ability Index, with a mean pre-pregnancy value of 1.15±0.36 versus a mean post-pregnancy value of 1.43±0.53. This change was statistically significant (p = 0.0001).

The mean pre-and post-pregnancy values of NYHA class and Ability Index just after the first pregnancy were 1.82±0.392 vs. 1.424±0,614 (p = 0.0046) and for Ability Index 1.091±0.292 vs. 1.394±1.197 (p = 0.0004); after the second pregnancy (N = 15), NYHA class and Ability index, unchanged (1.20±0.41 to 1.33±0.48; p = 0, 08; 1.267±0.458 to 1.400±1.119; p = 0.167).

#### Differences between pluriparous and nulliparous

The sub-analysis conducted in women with a history of two or more pregnancies compared to nulliparous patients showed a statistically significant difference concerning astenia (p = 0.034, OR 4.071); at echocardiography examination in the degree of right atrial dilatation (p = 0.000, OR 1.988), in fractional shortening and ejection fraction of right ventricular (p = 0.017, OR 0.931, p = 0.043, OR 0.949), in the degree of RV dysfunction (p = 0.008, OR 1.946), in the estimation of RV pulmonary pressure (0.000, OR 1.045) and in the degree of pulmonary hypertension (p = 0.005, OR 1.827).

While not reaching statistical significance, there was a probable statistical difference between nulliparous and multiparous in palpitations (p = 0.096, OR 2.734), in the duration of QT interval at surface electrocardiogram (p = 0.063, OR 0.937), in the results of late potentials electrocardiographic (p = 0.095, OR 1.801), in the severity of RV dilation (p = 0.074, OR 1.019), with higher values in multiparous women compared with nulliparous women. Logistic analysis for the research of independent predictors of adverse evolution, showed no statistically significant differences between nulliparous and multiparous.

## Discussion and Conclusions

The first surgical correction of TOF took place on 31 August 1954 by Lillehei. Surgery [1] has changed substantially the history of these patients: before surgery only a few patients, without the intervention reached adulthood. Currently more than 85% of patients with TOF is adult, representing the largest group in the GUCH community. However, it is also a group that needs a careful follow-up because of increasing incidence of complications, with aging.

A more precise distinction between sex and gender has recently emerged [10]. Sex is defined by biological, genetic, anatomical and physiological. Gender includes rather more complex dimensions related to the psychological, social, cultural, that make up the specificity of being men or women in a particular context.

In the past, when life expectancy of many patients with congenital heart disease were extremely poor, gender difference was between males and females, rather than between men and women, without considering the biology and anthropology.

From the end of the seventies, the improvement of diagnostic and therapeutic centers of Cardiology and Pediatric Cardiac Surgery helped many patients with congenital heart disease to grow up, attend school, become adolescents, are employed and generate children. About 40% of the community of adults CHD are women [11].

The distribution of diseases in patients of both sexes under 20 years of age, collected at Brompton Hospital Guch Unit in London,is comparable to that of children; therefore the relationship between gender and type of heart disease does not change with age, suggesting that gender does not appear to affect survival into adolescence and young adulthood [12].

For women with congenital heart disease adolescence involves the specificities related to the onset of menarche, to the ability to procreate, to sexuality. In the past most problems affecting quality of life were reported by women with congenital heart disease: the highest percentage of single and divorced, trouble finding a job suitable to their abilities, less capacity to accept the presence of physically disfiguring scars or skeletal variations of the thorax.

On the contrary our female Tetralogy of Fallot patients showed no difference with males about the level of education, since the same percentage have obtained a high school diploma, attended courses and obtained a university degree and were equally occupied; women prevailed in the choice of part-time job.

The pregnancy hasn't made the difference compared to nulliparous women. About 60% of the population is married; the group of women in pregnancy makes a difference, as more singles are found among those who have not been pregnant.

The development of programs of follow-up with the involvement of other specialists such as gynecologists, psychologists, and the strengthening of social services let the differences between the sexes in terms of quality of life to be reduced significantly, and offer the same odds of survival and better control of complications and sequela.

As confirmed in previous studies, the history of post-surgical women seems to be characterized by a favorable morpho-functional condition of the two ventricles, especially with less impairment of the left ventricle, compared with the same number of patients who had an anastomosis subclavian-pulmonary before the correction and for a period comparable to that of men [13].

In female patients the aortic valve was found less frequently insufficient and not in such severe degree as to require replacement with implants as it happened in some male patients.

Female patients presented more frequently required extracardiac surgery (not only gynecology) but they had less need of men and, more belatedly, of reoperations for failure of the pulmonary valve or residual shunt at the level of patch closure of the interventricular septum defect (in the two sexes has been used the same ventriculotomic trans-epicardial approach). Women complained more frequently fatigue and palpitations, however, most of them were in the same functional classes of men.

They used, in the same proportion of men, antiarrhythmic drugs, diuretics, antiplatelet drugs or anticoagulants, with a significant difference in the use of ACE inhibitors in men and greater consumption of proton pump inhibitors and anxiety in women. On the other hand, more women than men presented psychological disorders.

Just over one quarter of our patients presented severe ventricular arrhythmias, without a particular preference for one sex. The incidence of severe arrhythmias increased with follow-up time particularly in pregnant women. The number of patients who received a defibrillator is not significantly different, although males underwent to implant at a significantly younger age. For all groups dilatation and reduced ventricular ejection fraction, LV hypertrophy and late positive potential risk factors have proved as independent predictors of major arrhythmic events. The mean QRS duration of the entire male population is higher than the mean value of the female, in agreement with mean values of RV size higher in men (report mechano-electrical), but it assumes greater predictive value of arrhythmia when it is accompanied by left ventricular dysfunction and surgical history in the presence of a palliative operation.

These patients may support a pregnancy [14] while presenting a greater risk of losing the fetus, increased occurrence of malformations and, albeit rarely, of experiencing adverse events [15] such as heart failure and arrhythmias in the presence of LV dysfunction [16], pulmonary hypertension or severe pulmonary and/or tricuspid valve insufficiency with reduced contractile function of the RV. Naguib and Gatzoulis [17] reported that the hemodynamic load (specific during pregnancy), associated with cardiac structural changes (specific for correction surgery), cause 7% of all complications, particularly expansion and reduced RV function, with increased incidence of hyperkinetic ventricular or supraventricular arrhythmias; moreover the off-spring events happen more frequently during pregnancies in women with TOF who take medications for heart disease.

More than half of our patients had the experience of pregnancy, often unique, rare double and exceptionally triple, and the majority have completed at least one of their pregnancies, with an mean age lower than that of Italian women. However an high proportion of them experienced at least one complication including threatened abortion, miscarriage, placental abruption, preterm delivery. This confirms the high miscarriage (14.7% in TOF population versus 9.6% in general Italian population), which occurred always within the first 13 weeks of gestation. All pregnant women who completed pregnancy were in good functional condition, but after pregnancy there was a significant shift from first to second class both using NYHA functional class and Ability Index. Pregnant women with a unique pregnancy showed no significant differences in terms of morpho-functional features of ventricular cavity compared to nulliparous women, but they complained most frequently fatigue and palpitations, and echocardiography showed frequently mild and moderate degrees of tricuspid regurgitation with an enlarged right atrium. Fatigue was more pronounced in women with multiple pregnancies; these women compared with nulliparous had a more marked right atrial dilatation, a dilated right ventricle, limited pump ability and increased pulmonary arterial pressure. The recurrence in the offspring of a congenital heart defect was high, confirming that it is a concrete risk that must be explained to a woman who is planning to become pregnant and therefore the need to undergo an investigation using fetal echocardiography. In our series Caesarean interventions were frequently used, chosen by obstetricians and anesthesiologists as security. However natural childbirth, assisted, remains the most convenient choice; when it has happened, it doesn't cause any particular problems, only antibiotic prophylaxis for bacterial endocarditis, and electrical monitoring.

In conclusion there were no gender-specific differences in patients operated for Tetralogy of Fallot using ventriculotomy. For both, long-term results are satisfactory, although sequelae and complications are present in post-surgical follow-up and they get worse during time. Pregnancy is an event in these patients at risk for the newborn, in terms of miscarriage, prematurity, and recurrence of birth defects, and for the mother in terms of ventricular dysfunction and electrical instability.

However, at least a single pregnancy does not appear to significantly modify the natural history of post-surgical patients operated for Tetralogy of Fallot. The age of first pregnancy was spontaneously anticipated from our patients, compared to the indices reported by national statistical institutes and it's because time is not in their favor. Pregnancy is an integral part of congenital heart disease for women to live the “normality” obtained with the aid of surgical correction: this is why they are willing to take risks will be the responsibility of medical institutions to support this project and no life.

## References

[1] Lillehei CW, Cohen M, Warder HE, Read RC, Aust JB, et al (1955) “Direct vision inracardiac surgical correction of the tetralogy of Fallot and pulmonary atresia defect: report of first,ten cases. Ann Surg. 142: 418. 10.1097/00000658-195509000-00010 PMC1465089 13249340

[2] Nollert G, Fischlein T, Bouterwek S, Bohmer C, Klinner W, et al (1997) Long-term survival in patients with,repair of tetralogy of Fallot: 36-year follow-up of 490 survivors of the first year after surgical repair. J Am Coll Cardiol 30(5): 1374–83. 9350942 10.1016/s0735-1097(97)00318-5

[3] Gatzoulis MA, Balaji S, Webber SA, Siu SC, Hokanson JS, et al (2000) Risk factors for arrhythmia and sudden cardiac death late after repair of tetralogy of Fallot: a multicentre study. Lancet 356(9234): 975–81. 11041398 10.1016/S0140-6736(00)02714-8

[4] Veldtman GR, Connolly HM, Grogan M, Ammash NM, Warnes CA, et al (2004) Outcomes of pregnancy in women with tetralogy of Fallot. J Am Coll Cardiol 44: 174–80. 15234429 10.1016/j.jacc.2003.11.067

[5] Daliento L, Mapelli D, Russo G, Scarso P, Limongi, et al (2005) Health related quality of life in adults with repaired tetralogyof Fallot: psychosocial and cognitive outcomes. Heart 91: 2013–218. 10.1136/hrt.2003.029280 PMC1768729 15657236

[6] Shinebourne EA, Anderson RH. Fallot's tetralogy (2002) In:Paediatric cardiology. Anderson RH, Baker EJ, Macartney FJ, et al. Eds. 2nd. Toronto: Churchill Livingstone, London. 1213–502.

[7] Sarikouch S, Koerperich H, Dubowy KO, Boethig D, Boettler P, et al.. (2011) Impact of Gender and Age on Cardiovascular Function Late After Repair of,Tetralogy of Fallot: Percentiles Based on Cardiac Magnetic Resonance, Circ Cardiovasc Imaging. doi: CIRCIMAGING.111.963637. 10.1161/CIRCIMAGING.111.963637 21908707

[8] Lang RM, Bierig M, Devereux RB, Flachskampf FA, Foster E, et al (2005) Recommendations for Chamber Quantification: A Report from the American Society of Echocardiography's Guidelines and Standards Committee and the Chamber Quantification Writing Group, Developed in Conjunction with the European Association of Echocardiography, a Branch of the European Society of Cardiology J Am Soc Echocardiogr. 18: 1440–1463. 10.1016/j.echo.2005.10.005 16376782

[9] STATA (Stata Corp 2009: State Statistical Software: release 11.College, Station, TX: StataCorp LP).

[10] Rhoda UK (1979) Toward a redefinition of sex and gender. Am Psychologist 34(11): 1085–1094.

[11] Warnes CA, Liberthson R, Danielson GK, Dore A, Harris L, et al (2001) Task force 1: the changing profile of congenital heart disease in adult life. JACC 37(5): 1170–75. 11300418 10.1016/s0735-1097(01)01272-4

[12] Somerville J (1998) The Denolin Lecture: The woman with congenital heart disease. Eur Heart J 19(12): 1766–75. 9886718 10.1053/euhj.1998.1204

[13] Broberg CS, Aboulhosn J, Mongeon FP, Kay J, Valente AM, et al (2011) Prevalence of left ventricular systolic dysfunction in adults with repaired Tetralogy of Fallot. Am J Cardiol 107(8): 1215–20. 21349477 10.1016/j.amjcard.2010.12.026

[14] Veldtman GR, Connolly HI, Grogan M, Ammash NM, Warnes CA, et al (2004) Outcomes of pregnancy in women with tetralogy of Fallot. JACC 44: 174–80. 15234429 10.1016/j.jacc.2003.11.067

[15] Balci A, Drenthen W, Mulder BJ, Roos-Hesselink JW, Voors AA, et al (2011) Pregnancy in women with corrected tetralogy of Fallot: Occurrence and predictors of adverse events. Am Heart J 161: 307–13. 21315213 10.1016/j.ahj.2010.10.027

[16] Uebing A, Arvanitis P, Wei Li W, Diller GP, Babu-Naravan SV, et al (2010) Effect of pregnancy on clinical status and ventricular function in women with heart disease. Int J Card 139: 50–59. 10.1016/j.ijcard.2008.09.001 18835051

[17] Naguib MA, Dob DP, Gatzoulis MA (2010) A functional understanding of moderate to complex congenital heart disease and the impact of pregnancy. Part II: tetralogy of Fallot, Eisenmenger's syndrome and the Fontan operation. Int J Obstet Anesth 19(3): 306–12. 20627686 10.1016/j.ijoa.2009.10.009

